# Community-Based Restaurant Interventions to Promote Healthy Eating: A Systematic Review

**DOI:** 10.5888/pcd12.140455

**Published:** 2015-05-21

**Authors:** Jennifer N. Valdivia Espino, Natalie Guerrero, Natalie Rhoads, Norma-Jean Simon, Anne L. Escaron, Amy Meinen, F. Javier Nieto, Ana P. Martinez-Donate

**Affiliations:** Author Affiliations: Jennifer N. Valdivia Espino, Natalie Guerrero, Natalie Rhoads, Norma-Jean Simon, F. Javier Nieto, University of Wisconsin–Madison, Madison, Wisconsin; Anne L. Escaron, University of California, Los Angeles, California; Amy Meinen, University of Wisconsin–Madison and Wisconsin Obesity Prevention Network, Madison, Wisconsin.

## Abstract

**Introduction:**

Eating in restaurants is associated with high caloric intake. This review summarizes and evaluates the evidence supporting community-based restaurant interventions.

**Methods:**

We searched all years of PubMed and Web of Knowledge through January 2014 for original articles describing or evaluating community-based restaurant interventions to promote healthy eating. We extracted summary information and classified the interventions into 9 categories according to the strategies implemented. A scoring system was adapted to evaluate the evidence, assigning 0 to 3 points to each intervention for study design, public awareness, and effectiveness. The average values were summed and then multiplied by 1 to 3 points, according to the volume of research available for each category. These summary scores were used to determine the level of evidence (insufficient, sufficient, or strong) supporting the effectiveness of each category.

**Results:**

This review included 27 interventions described in 25 studies published since 1979. Most interventions took place in exclusively urban areas of the United States, either in the West or the South. The most common intervention categories were the use of point-of-purchase information with promotion and communication (n = 6), and point-of-purchase information with increased availability of healthy choices (n = 6). Only the latter category had sufficient evidence. The remaining 8 categories had insufficient evidence because of interventions showing no, minimal, or mixed findings; limited reporting of awareness and effectiveness; low volume of research; or weak study designs. No intervention reported an average negative impact on outcomes.

**Conclusion:**

Evidence about effective community-based strategies to promote healthy eating in restaurants is limited, especially for interventions in rural areas. To expand the evidence base, more studies should be conducted using robust study designs, standardized evaluation methods, and measures of sales, behavior, and health outcomes.

## Introduction

The Social Ecological Model posits that health results from interactions between individual and environmental factors (1). The food environment, defined as access to, availability of, and affordability of food (2), changed during the last century. Increases in portion size (3), availability of fast food (4), and fast food advertising (5) contributed to greater calorie consumption. These changes, paired with a more sedentary lifestyle (6), increased snacking (6,7), and more eating away from home (8) contributed to the rise in overweight and obesity rates. Since the 1970s, Americans increased their away-from-home share of calorie intake from fast food and table service restaurants, which are associated with higher caloric intake of foods high in fat and low in fiber, calcium, and iron (9). Thus, restaurants are important settings for interventions to improve the food environment.

The purpose of this review was to inform research led by community, academic, or local public health partnerships targeting restaurants. We aim to describe previous interventions and identify the level of evidence associated with different interventions. In defining “community-based restaurant intervention,” we adhere to the distinction between the community nutrition environment (encompassing the type, location, and accessibility of food outlets in a geographic community) and the organizational nutrition environment (encompassing food eaten, for example, at school or work). We focus on restaurants in the community, because access to restaurants inside organizational institutions is restricted to a subset of the public (2,10). The scope of the review is also limited to nonpolicy interventions voluntarily adopted by restaurant owners, because policy implementation is a long and cumbersome process. Although policy change is important, it may not always be feasible in a short term. For that reason, we studied interventions voluntarily adopted by restaurants to improve the food environment and promote healthy eating.

## Methods

### Data sources and study selection

We used the following search terms to search for English-language peer-reviewed journal articles from all years of PubMed and Web of Knowledge through January 2014: restaurant intervention, fast-food intervention, dining intervention, healthy choices, healthy eating, healthful eating, healthy dining, and mindful eating. Public health professionals provided a list of authors to search with these additional terms: environment, purchase, table service, promotion, and campaign. These searches yielded an initial 770 articles. After removing duplicates, 740 articles remained. The references and cited lists of articles were examined for other relevant studies, yielding an additional 20 relevant articles.

**Figure 1:**
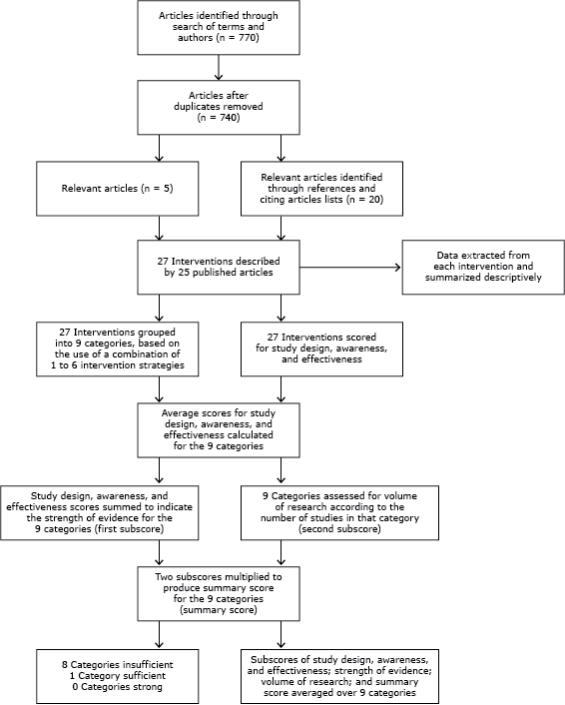
Methods to select studies, extract data, and describe and assess the level of evidence for community-based restaurant interventions to promote healthy eating, United States, 2014.

We judged the relevance of each study on the basis of the title and abstract and then read the articles deemed relevant for our review. Articles were included if they described original research about a voluntarily adopted health promotion intervention in one or more fast food or table service restaurants in community settings (ie, not located within school or work cafeterias). A total of 27 interventions described in 25 studies published from 1979 through 2013 met the inclusion criteria and were included in the review (Figure 1).

### Data extraction

#### Descriptions of interventions

Previous research (11) identified 6 restaurant intervention strategies: point-of-purchase information (POP), promotion and communication (Promotion), increased availability of healthy choices (Availability), reduced prices and coupons (Pricing), catering policies (Catering), and increased access (Access). Interventions that use POP highlight healthy choices, based on nutrition criteria, on a menu, menu board, or sign. Interventions that use Promotion use banners, table tents, or advertising in print or other media to promote healthy choices. Interventions that use Availability add healthy choices to the menu or modify menu items to make them healthier. Interventions that use Pricing offer special discounts or coupons to encourage healthy food purchases. The Catering strategy requires that healthy choices be offered at catered events. Interventions that use Access make healthy choices easier to locate or bring options to the public via food wagons (11). We grouped the interventions found during our review into intervention categories based on their use of any of the above strategies alone or in combination (eg, the category of POP + Promotion contained interventions that used both strategies together).

Two study authors (J.V.E. and N.G.) independently extracted data from each study (Table 1) and compared data to ensure consistency. Discrepancies were resolved through discussions among all the authors.

**Table 1:** Characteristics of 27 Community-Based Restaurant Interventions, Published Through January 2014

Characteristic	N (%)
**Country of intervention**	
United States	21 (77.8)
Canada	5 (18.5)
Netherlands	1 (3.7)
**US region**	
Northeast	3 (14.3)
South	7 (33.3)
Midwest	4 (19.0)
West	7 (33.3)
**Urbanicity of intervention locations** a	
Urban area	23 (85.2)
Urban cluster	1 (3.7)
Rural	1 (3.7)
Urban and urban cluster locations	1 (3.7)
Not reported	1 (3.7)
**Guiding theories, models, or approaches** b	
Health belief model	3 (11.1)
Matching theory	3 (11.1)
Social marketing	1 (3.7)
Theory of reasoned action	1 (3.7)
Asset-based community development	1 (3.7)
Community-based participatory research	1 (3.7)
Social cognitive theory	1 (3.7)
Planned behavior theory	1 (3.7)
Not reported	19 (70.4)
**Intervention strategies implemented** c	
Point-of-purchase information (POP)^d^	21 (77.8)
Promotion and communication (promotion)^e^	21 (77.8)
Increased availability (availability)^f^	17 (63.0)
Reduced prices and coupons (pricing)^g^	6 (22.2)
Catering policies (catering)^h^	0
Increased access (access)^i^	0
**Number of participating restaurants, outlets** j	
1	8 (29.6)
2–4	4 (14.8)
5–10	5 (18.5)
11–30	5 (18.5)
>30	5 (18.5)
**Duration of interventions** k	
≤1 month	9 (33.3)
>1– ≤6 months	6 (22.2)
>6 months– ≤1 year	3 (11.1)
>1 year	8 (29.6)
Not reported	1 (3.7)
**Main outcome measures** l	
Sales data^m^	19 (70.4)
Parton’s reported behaviors^n^	14 (51.9)
Theoretical mediators of behavior^o^	20 (74.1)
**Effectiveness** p	
Increase in outcome measures of 1%–25%	6 (22.2)
Increase in outcome measures of 26%–69%	6 (22.2)
Increase in outcome measures >70%	3 (11.1)
No change in outcome measures	4 (14.8)
No information about magnitude of change	8 (29.6)

a Categories of urbanicity were urban areas (population >50,000), urban clusters (2,500–50,000 residents), and rural areas (<2,500 residents) (12).

b Values represent the number of interventions that cited the specified theory, model, or approach. The cells do not sum to 27 or 100% because interventions cited multiple theories. The health belief model (13,14) and matching theory (15,16) were referred to in a study that produced 3 of the interventions examined (17). Social marketing (18) and the theory of reasoned action (19) were cited in a study (20). The following theories were cited once in 4 separate studies: asset-based community development (21,22), community-based participatory research (23,24), social cognitive theory (25,26), and the theory of planned behavior (27,28).

c Values represent the number of interventions that used the specified strategy. The cells do not sum to 27 or 100% because many interventions used a combination of strategies.

d Point of purchase interventions specified healthy choices on a menu, menu board, or sign (11).

e Promotion interventions use banners, table tents, or advertising to promote healthy choices (11).

f Availability interventions add healthy choices to the menu or modify menu items to make them healthier (11).

g Pricing interventions offer discounts or coupons to encourage healthy purchases (11).

h Catering interventions require healthy choices be served at catered events (11).

i Access interventions make healthy choices easier to locate (11).

j Number of restaurants participating at the time of evaluation. Median = 7 outlets, interquartile range (IQR) = 1–19.5 outlets, range = 1–222 outlets, mean = 25.96 outlets.

k Greatest number of weeks that the intervention lasted in at least 1 restaurant. Median = 10 weeks, IQR = 4–79 weeks, range = 1–260 weeks, mean = 50.27 weeks.

l Values represent the number of interventions that measured the specified outcome. The cells do not sum to 20 or 100% because many interventions measured multiple outcomes.

m Quantitative measures of food purchases.

n Measures of patrons requesting a menu item be prepared healthfully or consulting intervention materials in choosing meals.

o Measures of individuals’ awareness, knowledge, and intentions related to the intervention or healthy eating.

p Effectiveness is an intervention’s impact on the main outcome measures of sales data, reported behaviors, or theoretical mediators.

The main outcome measures reported were sales data (ie, quantitative measurement of purchases), patrons’ reported behaviors (eg, customers requesting an item be prepared healthfully or reporting that the intervention affected their order), and theoretical mediators of behavior change (eg, customers’ or community members’ awareness, knowledge, intentions). We summarized the interventions using descriptive summary statistics.

#### Assessment of evidence

We adapted a scoring system previously used to evaluate food environment interventions in supermarkets (29) and constructed based in part on the *Guide to Community Preventive Services* methods of systematic review (30) and the RE-AIM framework (31). Using this system, we assigned points to each intervention for each of 3 characteristics: study design, awareness, and effectiveness.

The study design score (0 to 3 points) reflects the ability of a study design to evaluate the effectiveness of the intervention (29). Interventions of the greatest suitability, defined as those with a “concurrent comparison group and prospective measurement” (30), were given 3 points (29). Interventions of moderate suitability, defined as those with “retrospective designs or multiple pre- or post-measurements but no concurrent comparison group,” (30) were given 2 points (29). Interventions of least suitability, defined as those with “single pre- and post-measurements and no concurrent comparison group or exposure and outcome measured in a single group at the same point in time,” (30) were given 1 point (29). Studies that did not describe study design or surveyed only restaurant personnel were assigned 0 points.

Awareness, or penetrance, scores (0 to 3 points) indicate the percentage of individuals surveyed (eg, restaurant patrons, community residents) who took note of intervention activities. Interventions were assigned 3, 2, or 1 point if they reported 70 to 100%, 26% to 69%, or 1% to 25% of the target audience were aware, respectively (29). Studies were assigned 0 points if awareness was 0% or if no measurement of awareness was reported. Awareness scores replace Reach scores in the original scoring (29,31), as awareness more specifically represents what the studies reported.

Effectiveness scores (0 to 3 points) reflect the intervention’s effect on the main outcome measures of sales data, reported behaviors, or theoretical mediators (29). Interventions were assigned 3, 2, or 1 point if they reported an improvement of 70% or more, 26% to 69%, or 1% to 25% in outcome measures associated with the intervention, respectively (eg, an intervention with a 50%, or 1.5-fold, increase in sales of healthy items at postmeasurement compared with premeasurement was assigned 2 points). Interventions were given 0 points if there was no difference between groups or between pre- and post-measurements (29). Interventions for which there was no quantitative information about the magnitude of effectiveness were assigned 0 points.

A difference between our scoring and the original scoring system (29) is that, for each of the dimensions listed above, we assigned 0 points to studies with missing information. When a study reported data by outlet (32–36), subgroup of the population (37,38), or follow-up time (26), we scored the intervention on the basis of average awareness and effectiveness. After assigning points to each intervention, average study design, public awareness, and effectiveness scores were computed for each of the categories. We then summed the average study design, public awareness, and effectiveness scores for each category to generate an indicator of the *strength of evidence* (first subscore), which has a possible range of 0 to 9 points, with higher scores representing stronger evidence levels (29).

Because replication is an important element of the scientific method, each category was assigned a *volume of research* score (second subscore, 1 to 3 points) reflecting the number of times these intervention categories have been replicated (29). Categories replicated 8 to 25 times were given 3 points; 2 to 7 times were given 2 points; only 1 study was given 1 point. The cutoffs were proportionally reduced from the original scoring because this review examined fewer studies (27 vs 33).

The summary score for each intervention category is the product of the 2 subscores (ie, strength and volume of research [Figure 1]), with a possible range of 0 to 27. Categories with summary scores of 0 to 9, 10 to 18, or 19 to 27 points were classified as having insufficient, sufficient, or strong evidence, respectively (29,30). These point cutoffs were again proportionally reduced from the original scoring because fewer studies were reviewed. To evaluate the overall level of evidence across all categories (ie, evidence for all restaurant interventions included in the review), the summary scores for all intervention categories was summed and divided by the number of categories (29).

## Results

### Description of interventions

**Figure 2:**
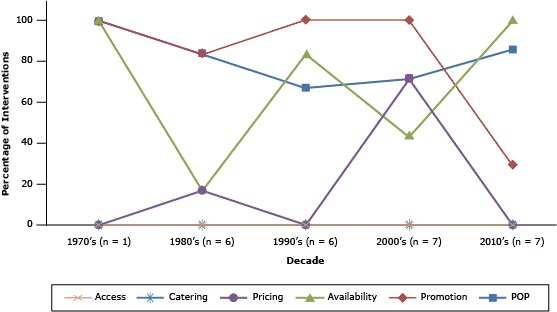
Percentage of interventions, within each decade, that implemented the following strategies: point-of-purchase information (POP), promotion and communication (Promotion), increased availability (Availability), reduced prices and coupons (Pricing), catering policies (Catering), and increased access (Access [11]). Data from 27 interventions, described in 25 reports of studies published through January 2014. **Table:** 

Of the 27 community-based restaurant interventions, 21 (77.8%) took place in the United States, and among them, most were conducted in the West (20,22,24,28,32,35,36) or South (17,39–42) (Table 1). Most (n = 23, 85.2%) interventions took place in urban areas. Eight interventions (29.6%, data not shown), described in 6 studies (17,20,22,24,26,28), were explicitly guided by health promotion theories, models, or approaches. The median number of participating outlets was 7 (range: 1–222 restaurants), and the median duration of the interventions was 10 weeks (range: 1–260 weeks). The most popular intervention strategies, used alone or in combination with other strategies, were POP (n = 21, 77.8%) and Promotion (n = 21, 77.8%), followed by Availability (n = 17, 63.0%) and Pricing (n = 6, 22.2%). We found no interventions that used Catering or Access. The distribution of strategies used over time is shown in Figure 2.

Decade (n)	Intervention Strategies, %					
	POP	Promotion	Availability	Pricing	Catering	Access
1970s (1)	100.00	100.00	100.00	0	0	0
1980s (6)	83.33	83.33	16.67	16.67	0	0
1990s (6)	66.67	100.00	83.33	0	0	0
2000s (7)	71.43	100.00	42.86	71.43	0	0
2010s (7)	85.71	28.57	100.00	0	0	0

#### Assessment of evidence

Of the 27 interventions, 15 (55%) reported improvements in at least 1 measured outcome: 6 (22.2%) reported an increase of 1% to 25%, 6 (22.2%) reported an increase of 26 to 69%, and 3 (11.1%) reported an increase of more than 70% in main outcomes. Four interventions (14.8%) reported no change in main outcomes, and 8 (29.6%) did not provide enough information to determine changes in outcomes. No intervention had an average negative impact on main outcome measures.

The 27 interventions fell within 9 categories. Table 2 shows the average scores obtained by each category. Studies included in each category are described in detail in Table 3. For all categories combined average scores were as follows: study design, 1.80 (range: 0–3); awareness, 0.60 (range: 0–1.83); effectiveness, 1.05 (range: 0–3); strength of evidence, 3.45 (range: 0–6); and volume of research, 1.67 (range: 1–2). The overall summary score for all categories combined was 5.76 (range: 0–10). POP + Availability (n = 6) was the only category that had sufficient evidence, based on our scoring system. This category had the highest average awareness score (mean = 1.83), moderately suitable study designs (mean = 1.83), and little missing information compared with other categories. Five of the 6 interventions reported improvements up to 70% in sales data, reported behaviors, or theoretical mediators, yielding a mean effectiveness subscore of 1.33.

**Table 2:** Average Scores for Community-Based Restaurant Interventions by Category, Published Through January 2014

Intervention Category^a^	Study Design (0–3)^b^	Awareness (0–3)^c^	Effectiveness (0–3)^d^	Strength of Evidence (0–9)^e^	Summary Score (0–27)^f^	Level of Evidence^g^
**Promotion (n = 1 intervention)**						
Points	3.00	0.00	0.00	3.00	3.00	Insufficient
**POP + Availability (n = 6 interventions)**						
Points	1.83	1.83	1.33	5.00	10.00	Sufficient
**POP + Promotion (n = 6 interventions)**						
Points	1.83	1.00	1.00	3.83	7.67	Insufficient
**Promotion + Availability (n = 3 interventions)**						
Points	1.00	1.33	1.00	3.33	6.67	Insufficient
**Promotion + Pricing (n = 1 intervention)**						
Points	3.00	0.00	3.00	6.00	6.00	Insufficient
**POP + Availability + Promotion (n = 5 interventions)**						
Points	1.00	0.20	0.60	1.80	3.60	Insufficient
**POP + Promotion + Pricing (n = 2 interventions)**						
Points	1.50	0.00	1.50	3.00	6.00	Insufficient
**Promotion + Availability + Pricing (n = 1 intervention)**						
Points	3.00	1.00	1.00	5.00	5.00	Insufficient
**POP + Availability + Promotion + Pricing (n = 2 interventions)**						
Points	0.00	0.00	0.00	0.00	0.00	Insufficient
**All 9 categories averaged**						
Points	1.80	0.60	1.05	3.45	5.76	Insufficient

a Categories represent the use of the following 6 intervention strategies singly or combined: promotion and communication (Promotion), point-of-purchase information (POP), increased availability (Availability), reduced prices and coupons (Pricing), catering policies (Catering), and increased access (Access [11]).

b Scored 0 to 3 points on the basis of the ability of a study design to evaluate effectiveness. Higher scores indicate stronger study design (29). Studies that did not describe the methods used to evaluate effectiveness or only surveyed restaurant owners and employees were assigned 0 points.

c Scored 0 to 3 points, indicates the percentage of surveyed individuals who noticed intervention materials with higher scores indicating a greater percentage reporting awareness. Interventions were assigned 3, 2, or 1 point if they reported 70% to100%, 26% to 69%, or 1% to 25% of the target audience were aware, respectively (29). Studies were assigned 0 points if awareness was 0% or if no measurement of awareness was reported.

d Scored 0 to 3 points, with higher scores indicating a greater impact on intervention’s main outcome measures of sales data, reported behaviors, or theoretical mediators. Interventions were assigned 3, 2, or 1 point if they reported a ≥70%, 26% to 69%, or 1% to 25% improvement in outcome measures associated with the intervention (29).

e Strength of evidence score has a possible range from 0–9 and is the sum of the average study design, awareness, and effectiveness scores for each category. Higher scores indicate stronger evidence levels (29).

f The summary score is the product of the strength of evidence score and the volume of evidence score. The category is assigned a volume of research score of 1–3 points according number of interventions in each category, with higher scores indicating more interventions within that category. Categories including 8 to 25 interventions were given 3 points. Categories including 2 to 7 interventions were given 2 points. Categories including only 1 intervention were given 1 point. The summary score has a possible range of 0 to 27 (29).

g Categories with scores of 0–9, 10–18, or 19–27 points have insufficient, sufficient, or strong evidence, respectively (29,30).

**Table 3:** Studies Published from 1979 Through January 2014 on Community-Based Restaurant Interventions (n = 27) to Promote Healthy Eating, by Category^a^

Intervention Category^a^	Summary Data
**Promotion**	
**Wagner and Winett, 1988** (42)	
Setting, location, and urbanicity	1 intervention outlet and 1 control outlet, Blacksburg, Virginia; urban cluster
Activities and duration	Promoted eating low-fat salads with posters, table tents, banners, streamers, and computerized messages at registers; 4 weeks
Study design	Prospective measurement with comparison group
Public awareness	No information
Main outcome measures	Sales data of salads and nontarget menu items
Effectiveness	No significant difference between salad sales in intervention group than control group at posttest
**POP + Availability**	
**Paul et al, 1989** (40)	
Setting, location, and urbanicity	39 outlets, Richmond, Virginia, and Blue Ridge, Virginia; urban area and urban cluster, respectively
Activities and duration	Created and labeled American Heart Association–approved items on the menu; no duration information
Study design	Single postmeasurement; no comparison group
Public awareness	57% of patrons surveyed were aware of the intervention
Main outcome measures	Counts of reported willingness of patrons to try specific reduced-calorie menu items and assessment of healthy eating knowledge among patrons and restaurant staff
Effectiveness	Patrons surveyed were more likely to request healthy preparation of meat dishes than low-calorie desserts and entrées; no significant differences between patrons and restaurant staff in knowledge about healthy eating
**Pulos and Leng, 2010** (35)	
Setting, location, and urbanicity	18 outlets, Pierce County, Washington; urban area
Activities and duration	Created and labeled target menu items; 2 years
Study design	Multiple pre- and postmeasurements; no comparison group
Public awareness	71% of patrons surveyed reported noticing the nutrition information
Main outcome measures	Reported behavioral change because of program and sales data on calories sold
Effectiveness	33% of patrons surveyed reported they changed behavior after seeing nutrition information
**Chen et al, 2011** (24)	
Setting, location, and urbanicity	1 outlet, Seattle, Washington; urban area
Activities and duration	Educated owner about diabetes and nutrition; owner created healthy menu items and labeled them “lighter” options; promoted diabetes-friendly meals on menu; 6 weeks
Study design	Multiple postmeasures, no comparison group
Public awareness	No information
Main outcome measures	Sales data
Effectiveness	11.6% of entrees sold postintervention were from the lighter options menu
**Sharma et al, 2011** (28)	
Setting, location, and urbanicity	1 outlet, San Francisco Bay Area, California; urban area
Activities and duration	Created and modified menu items to meet the South Asian Heart Center and the National Cholesterol Education Program’s Therapeutic Lifestyle Changes guidelines; identified healthy items with a heart symbol; 11 weeks
Study design	Multiple pre- and postmeasurements; no comparison group
Public awareness	100% of a sample of adult customers noticed healthy menu items
Main outcome measures	Computerized sales data
Effectiveness	Sales increased by 9.9% on average; sales of 3 of the 9 target items saw increased from 38% to 75%
**Nevarez et al, 2013** (36)	
Setting, location, and urbanicity	7 outlets, South Los Angeles, California; urban area
Activities and duration	Created healthy menu items and posted calorie information on menu boards; restaurants developed brochures with detailed nutrient content; 2 years
Study design	Single postmeasurement; no comparison group
Public awareness	65% of adult patrons interviewed noticed nutrition information
Main outcome measures	Awareness and attitudes toward menu labeling and reported influence of the program
Effectiveness	46% of patrons interviewed reported that their purchases were influenced by calorie information
**Papies and Veling, 2013** (38)	
Setting, location, and urbanicity	1 outlet, Netherlands; unknown urbanicity
Activities and duration	Half of the restaurant menus were supplemented with diet-related words and the other half served as control menus; 3 weeks
Study design	Prospective measures with comparison group of customers with control menus
Public awareness	No information
Main outcome measures	Proportion of healthy menu choices ordered by intervention and control group
Effectiveness	The proportion of healthy menu choices was approximately 35% in the intervention group and 15% in the control group; intervention was more effective among dieters than nondieters
**POP + Promotion**	
**Colby et al, 1987** (43)	
Setting, location, and urbanicity	1 outlet, Pawtucket, Rhode Island; urban area
Activities and duration	Three menu items were promoted as daily specials with 3 alternating messages with different focuses: taste and health, health alone, and nonspecific focus (control); 9 weeks
Study design	Prospective measures with comparison group
Public awareness	No information
Main outcome measures	Sales data of the number of target items sold concordant with each message
Effectiveness	The number of items sold was not significantly different for the 3 messages
**Forster-Coull and Gillis D, 1988** (44)	
Setting, location, and urbanicity	18 outlets, Halifax, Canada; urban area
Activities and duration	Labeled menu items in compliance with Canadian dietary recommendations with heart stickers or listed them on a menu insert; provided suggestions to reduce fat intake with table tents; advertised program with certificates for the restaurant and flyers; held promotional luncheon for media and community leaders; 6 weeks
Study design	Single postmeasurement; no comparison group
Public awareness	70% of surveyed patrons could name 1 characteristic of the program’s dietary guidelines; 69% could list 1 or more items from the menu
Main outcome measures	Reported orders of program-approved menu items and reports of requests for sauces to be served on the side of an entree
Effectiveness	Reports of ordering a healthy lunch increased by 15% and reports of ordering sauce served on the side increased by14%
**McPharlin, 1988** (45)	
Setting, location, and urbanicity	More than 100 outlets, Bloomington, Rochester, and St. Paul, Minnesota; urban areas
Activities and duration	Labeled existing menu items with a heart symbol on the basis of criteria established by the Minnesota Nutrition Subcommittee of the American Heart Association; distributed posters to restaurants and brochures to patrons; 4 years
Study design	No information regarding design to evaluate effectiveness
Public awareness	No information
Main outcome measures	No information
Effectiveness	No information
**Albright et al, 1990** (32)	
Setting, location, and urbanicity	4 outlets, Northern California;, urban clusters
Activities and duration	Labeled menu items low in fat and cholesterol with a heart to indicate “good for health”; encouraged patrons to create healthy meals with tip sheets of suggestions; 4 weeks
Study design	Multiple pre- and postmeasurements; no concurrent comparison group
Public awareness	83.5% of patrons saw menu labels
Main outcome measures	Sales data on target items by outlet; reported behavior change after referring to tip sheet
Effectiveness	Sales increased by an average of 15.5% with 1 outlet witnessing an increase of 40%; on average, 53% of patrons followed 1 or more tips on the tip sheet; on average, 29.5% of patrons selected a labeled item
**Horgen and Brownell, 2002** (17)	
Setting, location, and urbanicity	1 outlet, Huntsville, Alabama; urban area
Activities and duration	Health messages (gain-framed and loss-framed) accompanied a list of healthy food options; 1 week
Study design	Prospective measurement with a comparison group of nontarget items
Public awareness	No information
Main outcome measures	Sales data on target and nontarget items
Effectiveness	Sales of target items increased by 201% on average compared with baseline; sales of nontarget comparison items remained constant
**Fitzgerald et al, 2004** (34)	
Setting, location, and urbanicity	9 outlets, Ann Arbor, Michigan; urban area
Activities and duration	Labeled “healthy dining” menu items on the basis of program and Food and Drug Administration criteria; promoted program with newspaper advertisement, posters, and table tents; 8 weeks
Study design	Multiple pre- and postmeasurements; no concurrent comparison group
Public awareness	No information
Main outcome measures	Electronic sales data recording the proportion of target items sold of all tracked items (a group of target and comparison items)
Effectiveness	No significant difference in proportion of sales of target items between pre- and posttest
**Promotion + Availability**	
**Green KL et al, 1993** (37)	
Setting, location, and urbanicity	130 outlets, Saskatoon and Regina, Saskatchewan, Canada; urban areas
Activities and duration	Recruited restaurants willing to provide healthy choices or preparations upon request; promoted the program with Heart Smart logo in restaurants and advertised in the *Heart and Stroke Foundation Dining Guide* for community members; 1.83 years
Study design	Single time point; no comparison group
Public awareness	22% and 41% of a sample of Regina and Saskatoon residents heard of the program; average awareness was 31.5%
Main outcome measures	Awareness and understanding of the program, self-reported frequency of healthy food requests, and frequency of restaurant accommodation of request
Effectiveness	6.4% and 3.6% of a sample of Regina and Saskatoon residents used the program; on average, 5% of community members made a healthy request
**Dwivedi and Harvey, 1999** (46)	
Setting, location, and urbanicity	222 outlets, municipality of Ottawa-Carleton, Canada; urban area
Activities and duration	Recruited restaurants willing to provide healthy choices or preparations on request; encouraged patrons to request healthy preparations with menu inserts, table tents, restaurant certificates, posters, and advertisements in newspapers; 1.23 years
Study design	Design not suitable to evaluate effectiveness
Public awareness	No information
Main outcome measures	Restaurant owners’ use of materials and thoughts about the program
Effectiveness	No customer data
**Nothwehr et al, 2013** (26)	
Setting, location, and urbanicity	4 outlets, small towns in Iowa; rural
Activities and duration	Recruited restaurants willing to provide healthy choices or preparations on request; encouraged patrons to request healthy preparation of menu items with table tents, and window signs; 1 year
Study design	Multiple pre- and postmeasurements; no comparison group
Public awareness	Average awareness for all 3 follow-ups was 68%
Main outcome measures	Awareness and use of the program
Effectiveness	34% of patrons surveyed reported the table tent affected their order
**Promotion + Pricing**	
**Horgen and Brownell, 2002** (17)	
Setting, location, and urbanicity	1 outlet, Huntsville, Alabama; urban area
Activities and duration	Promoted price reductions (20%–30%) of target items on boards at entryway and on menu; 3 weeks
Study design	Prospective measurement with a comparison group of nontarget items
Public awareness	No information
Main outcome measures	Sales data of target and nontarget items
Effectiveness	Sales of target items increased by 357% on average compared with baseline; sales of nontarget comparison items remained constant
**POP + Availability + Promotion**	
**Scott et al, 1979** (41)	
Setting, location, and urbanicity	2 outlets, Houston, Texas, USA; urban area
Activities and duration	Created and labeled menu items low in cholesterol and saturated fat; promoted the program in the newspaper; 1 year
Study design	Multiple postmeasures; no comparison group
Public awareness	No information
Main outcome measures	Sales data
Effectiveness	No significant change in sales over 12-month post intervention
**Anderson and Hass, 1990** (47)	
Setting, location, and urbanicity	53 outlets, Colorado locations, USA, unknown urbanicity
Activities^b^ and duration	Created and labeled menu items low in calories, fat, cholesterol, and sodium; table tents provided information about the program and encouraged patrons to try labeled items; 4 weeks
Study design	Multiple pre- and postmeasurements no comparison group
Public awareness	No information
Main outcome measures	Sales data
Effectiveness	52 out of the 58 target items had an increase in sales, but no information about the magnitude of the increase in sales was provided
**Fitzpatrick et al, 1997** (48)	
Setting, location, and urbanicity	9 outlets, Vancouver, British Columbia, Canada; urban area
Activities and duration	Created and labeled menu items with reduced fat and smaller portion sizes; promoted through local media, table tents, menu inserts, and window decals; 4 weeks
Study design	Single postmeasurement; comparison group of regular items
Public awareness	No information
Main outcome measures	Satisfaction with reduced-fat foods compared with regular items
Effectiveness	Overall customer satisfaction was higher when served lower-fat item (rated 4.5 out of 5), compared with satisfaction with a regular item (4.28 out of 5), resulting in a 5.1% difference
**Richard et al, 1999** (33)	
Setting, location, and urbanicity	2 outlets, St. Henri, Montreal, Quebec, Canada; urban area
Activities^b^ and duration	Created and labeled menu items; expanded menu to include lowfat milk and dressing, and whole wheat bread; promoted the program with posters, placemats, newspapers, and leaflets; 19 weeks
Study design	Single postmeasurement; no comparison group
Public awareness	Average awareness of the program was 23.6%
Main outcome measures	Reported behaviors
Effectiveness	On average, 53.4% of surveyed patrons ordered targeted entrées; specifically, 77.1% in family style restaurant and 18% in fast-food restaurant ordered targeted entrée
**Blair et al, 2011** (39)	
Setting, location, and urbanicity	6 outlets, Frederick County, Massachusetts USA; urban area
Activities^b^ and duration	Created and labeled menu items for a diabetes awareness month challenge; promoted challenge through table tents, flyers, newspapers, and radio stations; 4 weeks
Study design	Design not suitable to evaluate effectiveness
Public awareness	No information
Main outcome measures	General response from patrons and restaurant staff
Effectiveness	No information
**POP + Promotion + Pricing**	
**Lefebvre, 1986** (49)	
Setting, location, and urbanicity	26 outlets, Pawtucket, Rhode Island; urban area
Activities^b^ and duration	Labeled existing healthy menu items; promoted through table tents, cooking demonstrations, and advertising in newspapers and in the *Guide to Healthy Eating* for the public; coupons were available at some locations; 3.77 years
Study design	Design not suitable to evaluate effectiveness
Public awareness	No information
Main outcome measures	Restaurant owners’ response to the program
Effectiveness	No information
**Horgen and Brownell, 2002** (17)	
Setting, location, and urbanicity	1 outlet, Huntsville, Alabama; urban area
Activities and duration	Identified a list of healthy options with health messages (gain-framed and loss-framed); promoted price reductions (20%–30%) of target items on boards at entryway and on menu; 2 weeks
Study Design	Prospective measurement with a comparison group of nontarget items
Public awareness	No information
Main outcome measures	Sales data of target and nontarget items
Effectiveness	Sales of target items increased by 326% on average compared with baseline; sales of nontarget comparison items remained constant
**Promotion + Availability + Pricing**	
**Acharya et al, 2006** (20)	
Setting, location and urbanicity	9 or more outlets, Greater San Diego Area, California; urban area
Activities and duration	Created and promoted healthy menu items through table tents, posters, community events, and ads in magazines, newspaper, and television; distributed coupons; 1 year
Study design	Prospective measures with a comparison group of restaurants
Public awareness	11.5% of patrons surveyed were aware of the Treat Yourself Well program
Main outcome measures	Beliefs and attitudes toward healthy options and reported purchase of a healthy menu item
Effectiveness	The intervention survey respondents were 3.7% more likely to purchase the healthy menu items than the control group; coupon-holders were 17% more likely to purchase a healthy item
**POP + Availability + Promotion + Pricing**	
**Economos et al, 2009** (50)	
Setting, location, and urbanicity	21 outlets, Somerville, Massachusetts; urban area
Activities^b^ and duration	Created and labeled menu items; promoted through table tents, menu inserts, signs, and newsletters; provided coupons; 1.62 years
Study design	Design not suitable to evaluate effectiveness
Public awareness	No information
Main outcome measures	Restaurant owners’ use of materials and thoughts about the program
Effectiveness	No information
**Hanni et al, 2009** (22)	
Setting, location, and urbanicity	16 or more outlets, Salinas, CA; urban area
Activities and duration	Created and labeled menu items; promoted through newspaper ads and brochures about diabetes risk assessment; coupons were provided; 5 years
Study design	Design not suitable to evaluate effectiveness
Public awareness	No information
Main outcome measures	Restaurant owners’ use of materials and thoughts about the program
Effectiveness	No information

Abbreviation: POP, point of purchase.

a Interventions were grouped into categories according to their use of the following intervention strategies singly or combined: promotion and communication (Promotion), point-of-purchase information (POP), increased availability (Availability), reduced prices and coupons (Pricing), catering policies (Catering), and increased access (Access)(11).

b This intervention clearly describes that individual restaurant owners had the flexibility to choose some or all of the strategies offered. Thus, the intervention category reflects the range of activities carried out by the intervention.

The remaining 8 categories had insufficient evidence. The 6 interventions in the POP + Promotion category (summary score = 7.67) had moderately suitable study designs (mean = 1.83), but only 3 of the 5 interventions that carried out effectiveness evaluations reported increases in main outcome measures (mean = 1.00). Furthermore, 4 interventions did not measure awareness, which lowered the average awareness score to 1.00. Two studies with the strongest study designs in this category had opposite findings (17,43). The intervention by Horgen and Brownell included the development of a list of healthy eating options along with health messages to encourage the selection of these items. The intervention was tested over an 8-day period in 1 restaurant in Huntsville, Alabama, in 2002. The study used a strong design with premeasures and postmeasures of sales of targeted and control menu items. The results showed that sales of targeted healthy menu items during the intervention compared with baseline increased on average by 200% and that the sales of control items did not change over the intervention period (17). The other intervention, by Colby et al, promoted 3 menu items using alternating messages that focused on 1) taste and health, 2) health only, and 3) unrelated topics (control). The intervention was tested in 1 restaurant in Pawtucket, Rhode Island, over a 9-week period. By using a strong study design that compared sales of items associated with each type of message the team found no differences in sales when comparing the 2 types of health messaging with nonspecific promotion of healthy daily specials (43).

The category of Promotion + Availability (n = 3) had insufficient evidence (summary score = 6.67). Two of the interventions (26,37) showed awareness from 26% to 69% and less than a 70% improvement in main outcome measures, resulting in mean awareness and effectiveness scores of 1.33 and 1.00, respectively. However, 1 of the 3 interventions in the category provided no information about study design, awareness, or effectiveness (46). A similar pattern of limited reporting and moderate effectiveness contributed to a low score for the category POP + Availability + Promotion (n = 5; summary score = 3.60). For the combination of strategies, POP + Availability + Promotion + Pricing (n = 2), the lack of suitable study designs and data regarding awareness and effectiveness resulted in a summary score of zero. In general, categories with 1 to 2 interventions had low scores.

## Discussion

Our review of community-based restaurant interventions indicates that the level of evidence available across intervention categories is still limited. After consolidating the evidence from all categories, we found insufficient evidence to suggest that community-based restaurant interventions were successful in promoting healthy eating. However, when examining each category of intervention, we did find some promising results based on our scoring system. For example, there was sufficient evidence to support the implementation of interventions that pair the strategies of POP + Availability. This category is represented by 6 interventions that implemented moderately strong study designs, reported public awareness, and demonstrated increases in main outcome measures.

The remaining 8 categories had insufficient evidence to suggest effectiveness according to our methods of assessment. Weak study designs and limited reporting of awareness and impact on outcomes were the main contributing factors for the low level of evidence supporting the use of these approaches. Low evidence levels for some categories were also explained by interventions that showed no or little effectiveness or interventions within the same category reporting mixed findings regarding effectiveness. Among the categories with insufficient evidence, 2, Promotion + Pricing and POP + Promotion + Pricing, showed the greatest promise. These categories included interventions with strong study designs and more than 200% improvements in measured outcomes (the highest among all studies included in this review). Despite these strengths, the categories contained only 1 or 2 interventions each, which resulted in low summary scores. These findings suggest that interventions combining these strategies may be effective and should continue to be tested in the future.

In systematically assessing the evidence, it was clear that within the same intervention category, intervention outlets, specific activities, messages, and materials varied substantially across interventions. For example, in some instances, the type of participating restaurants was not specified as fast food or family style, and not every outlet was included in the evaluation. Additionally, some activities and messages developed to promote healthy menu items may have been more effective than others. Variations in dosage and duration of the intervention also could have affected the reach and impact of the interventions.

Consequently, in interpreting the results of our review, it is important to consider not only the evidence estimated for each intervention category, but also individual interventions that have shown remarkable effects. Among these are the 3 interventions conducted by Horgen and Brownell. These interventions were tested with strong study designs and demonstrated a 2-fold to almost 4-fold increase in sales of healthy items (17). Similarly, the intervention by Papies and Veling, which used an equally strong study design, reported a 33% difference in the proportion of healthy menu choices ordered by those in the intervention group compared with the control group (38). Three other interventions showed moderate effectiveness and outstanding levels of public awareness (26,32,35).

Our review identified significant gaps in the literature. Many interventions lacked strong study designs. For example, 20 of the 27 interventions did not use comparison outlets or control menu items to evaluate the impact of the intervention on sales, patrons’ reported behaviors, or theoretical mediators (22,24,26,28,32–37,39–41,44–50). Of these, 8 interventions did not evaluate or did not report the magnitude of the intervention’s impact on the outcomes (22,39,40,45–47,49,50). Among the interventions that did report changes in outcome measures, the methods and instruments varied widely, making a comparison of effects across interventions and categories difficult. For example, some studies considered average daily sales, others measured weekly changes, and some interventions reported the number of healthy items with sales increases, but did not report the magnitude of these increases. Other studies relied on restaurant owners’ qualitative interpretations of the impact of the intervention, which could not be assessed with our scoring system (22,36,39,50). To expand the current evidence-base, standardized metrics must be created to evaluate restaurant interventions.

Additionally, few studies directly compared different intervention categories. The study by Horgen and Brownell was the only study that used similar methods to directly compare different intervention categories in the same restaurant (17). More comparative research of different categories of restaurant interventions needs to be conducted.

The evidence base regarding restaurant interventions would be further strengthened by more investigations outside primarily urban areas and additional data on distal outcomes. Little is known about the effectiveness of these intervention strategies in urban clusters and rural areas (26,32,40,42). There is also little information regarding short- or long-term health outcomes associated with these interventions. We envision these areas will be expanded as the use and study of restaurant interventions evolve in future years.

Our methods have limitations. The search for articles was limited to studies published in peer-reviewed journals. This method could have introduced publication bias if the published studies are not representative of all community-based restaurant interventions and especially if they are more likely to report successful interventions. Interventions that were successful may have been conducted in communities that were ready for behavior change and more responsive to the intervention (27,51). The search was conducted through electronic databases, which may have excluded older literature available only in hard copy or not cited by articles found through the online search.

Our scoring system has limitations. In essence, we averaged the effects of different programs with a varied number of outlets, different intervention durations, distinct definitions of healthy menu items, different comparison groups, and intervention-specific instruments to measure effects. Similar to the interventions in another review (52), many interventions in this review targeted menu items according to different criteria (eg, low-fat, low-calorie) and presented POP information differently (eg, stickers, menu inserts), which may have produced different effects. Interventions with different kinds of comparison groups (eg, control restaurant, menu items, community) were considered equal in the procedure to assign point values. Similarly, interventions that produced increases in self-reported behaviors or in the theoretical mediators of healthy eating were given the same point value as those interventions that produced substantial changes in sales data, a more objective outcome. Additionally, although flexibility in implementation may have enhanced intervention adoption, this could have diluted the strength scores for specific combined strategies because the effectiveness of the strategies offered to restaurants was assessed and not necessarily those implemented by each participating outlet.

Finally, our scoring system weighs more heavily those categories that have been evaluated by a higher number of studies, highlighting the importance of replication of interventions in assessing the evidence-base. With this system, newer or innovative combinations of strategies that have only been implemented in a few published studies cannot obtain high volume of research subscores. In turn, this influences the determination of insufficient evidence. As more evaluations of those intervention categories are published in the future, the scoring can be replicated to produce updated summaries of the levels of evidence associated with healthy eating interventions in restaurant settings. Finally, only 1 author (J.V.E.) assigned points to each intervention and conducted the scoring analysis. This author was in touch with the creator of the original scoring system (29) and the senior corresponding author (A.M.D.) to verify correct application of the methods.

Despite these limitations, this review summarizes relevant studies and identifies future areas of research on interventions in community restaurants. Although national policy changes, such as the menu-labeling regulation (53), are important to promote healthier diets on a population level, voluntary, local changes in the food environment can also contribute to this end. To combat obesity and its related health problems, health promotion professionals must be aware of the evidence regarding these intervention strategies. We encourage investigators to continue implementing and rigorously evaluating restaurant interventions, especially those showing sufficient evidence or promising success in promoting healthy eating.
